# Management of oral sub-mucous fibrosis using platelet-rich plasma and triamcinolone with hyaluronidase

**DOI:** 10.6026/973206300210599

**Published:** 2025-04-30

**Authors:** Ajay Kumar, Adit Srivastava, Neha Sah, Amlendu Shekhar, Sivani Darjee, Saumya Shukla, Vinayalekshmy IN Nair, Mahesh Khairnar, Ashutosh Kumar Rai

**Affiliations:** 1 Department of Oral medicine and radiology, Institute of medical sciences, Banaras Hindu University, Varanasi, Uttar Pradesh, India; 2 Department of Dentistry, Maa Vindhyavasini Autonomous State Medical College, Mirzapur, Uttar Pradesh, India

**Keywords:** Oral submucous fibrosis (OSMF), platelet-rich plasma (PRP), triamcinolone acetonide, hyaluronidase, randomized clinical trial

## Abstract

Oral Submucous Fibrosis (OSMF) is a chronic, progressive and potentially malignant disorder of the oral mucosa, primarily associated with betel quid and areca nut chewing. Therefore, it is of interest to compare the efficacy of intralesional Platelet-Rich Plasma (PRP) versus triamcinolone acetonide with hyaluronidase in the treatment of oral submucous fibrosis. This is a randomized clinical trial was conducted at the unit of oral medicine and radiology, Faculty of Dental Sciences, IMS, Banaras Hindu University (BHU), Varanasi. Patients diagnosed with grade II and III oral submucous fibrosis participated in this study. Results show that intralesional PRP was more effective than the combination of triamcinolone acetonide and hyaluronidase in the management of oral submucous fibrosis.

## Background:

The buccal mucosa is the mucous membrane lining the oral cavity. Oral submucous fibrosis (OSMF) is a chronic, progressive and precancerous condition. The main cause is chewing betel quid, areca nut, or both [3]. Oral sub mucous fibrosis is a chronic disease affecting the oral cavity, pharynx, or esophagus. It causes mucosal rigidity near the epithelial layers. As a result, mouth opening gradually becomes restricted [1]. Oral submucous fibrosis is mainly seen in India, Bangladesh, Sri Lanka, Pakistan, Taiwan and China, with a reported frequency ranging up to 0.4% in the Indian rural population [4]. In recent years, oral submucous fibrosis has increased in many parts of India. States like Bihar, Maharashtra, Gujarat and Madhya Pradesh are affected. Younger generations suffer more due to advertisements of areca nut products. Colorful pouches of gutkha and pan masala attract consumers. Areca nut is a key factor in the development of oral submucous fibrosis [2]. The copper content of areca nut is elevated and the levels of soluble copper in saliva may go up in areca quid chewers [5]. Several factors contribute to this condition, including chili consumption and nutritional deficiency. Hereditary susceptibility and autoimmunity are also suggested causes. The disease starts with vesicle formation, leading to inflammation and fibrosis. Hyalinization of the lamina propria causes thick fibrotic bands. These bands affect the cheeks, faucial pillars and lips. Trismus and tongue protrusion difficulty occur as a result. Reduced vascularity causes blanching of the oral mucosa. This gives the mucosa a marble-like appearance [6]. Dysphagia (difficulty in swallowing) and dysphonation (difficult phonation) are seen in advanced stages which results in malnutrition and poor oral hygiene [7]. Management depends on the severity of the disease. Stopping areca or betel nut chewing can resolve symptoms. Early diagnosis before fibrosis formation improves outcomes [8, 9]. Other treatments include physical therapy, medications and surgery. Corticosteroids are given through submucosal injections or topical application. Platelet-rich plasma (PRP) is an advanced wound therapy in dentistry [10, 11]. Platelet-rich plasma is the autologous component of the plasma derived from patient blood. It contains higher concentrations of platelets, chemokines, growth factors and cytokines [12]. PRP is made using a method called differential centrifugation. This process spins the blood at different speeds to separate its parts based on their weight [13] then injected into the oral submucosa. It induces platelets to release pro-inflammatory mediators and growth factors. These trigger wound healing and tissue remodeling. The acceleration of healing by PRP depends on the synergistic effects of these growth factors. Several glucocorticoids are used for oral submucous fibrosis treatment. These include hydrocortisone (short-acting), triamcinolone (intermediate-acting) and betamethasone or dexamethasone (long-acting) Steroids inhibit phospholipase A2 production, reducing prostaglandin and leukotriene synthesis. They stabilize lysosomal membranes and prevent the release of proteolytic enzymes. The combination of Triamcinolone Acetonide with Hyaluronidase is well-documented in the literature. However, the intralesional use of platelet-rich plasma is newer. No study has compared PRP with the Triamcinolone Acetonide and Hyaluronidase combination [14]. Therefore, it is of interest to compare the efficacy of intralesional Platelet-Rich Plasma (PRP) versus triamcinolone acetonide with hyaluronidase in the treatment of oral submucous fibrosis.

## Materials and Methods:

## Source of data:

The data were collected from patients reporting to the OPD of the unit of oral medicine and radiology, faculty of dental sciences, IMS, BHU and Varanasi. Patients were accepted who were clinically diagnosed with grade II and III oral submucous fibrosis (Clinical staging by Lai's 1995). And fulfill the inclusion and exclusion criteria. The study throughout 1year

## Ethical clearance:

The study was a randomized clinical trial conducted after obtaining ethical clearance from the Institutional Ethical Committee of the Faculty of Dental Sciences, IMS and BHU. The ethical noise is EC3454. The study was registered with the clinical trial registration number for this trial is CTRI/2022/10/046263. Each patient included was informed about the treatment protocol and informed consent was obtained.

## Method of collection of data:

[1] A simple random sampling method was used for this research.

[2] Sample size determination

## Patient selection criteria:

## Inclusion criteria:

[1] Age above the 17 years.

[2] Grade II and III oral submucous fibrosis Patients, who had not undergone any type of treatment for oral submucous fibrosis in the past 1 year.

[3] Burning sensation during eating spicy food.

[4] Presence of palpable fibrous bands on the buccal mucosa.

## Exclusion criteria:

[1] Who had received previous treatment for oral submucous fibrosis within 1 year

[2] Uncontrolled systemic illness patients.

[3] Any other mucosal/potential medical disorders

[4] History of drug allergy.

[5] Pregnant or breastfeeding women.

## Methodology and injections used:

Patients were randomly divided into two groups, 'A' and 'B', with 22 patients in each group (Figure 1 - see PDF). In group A, injection Triamcinolone Acetonide 40mg (1ml) with Hyaluronidase 1500IU combination filled in a 5ml syringe, was injected intralesional into the retro-molar trigone and in the fibrous band along the buccal mucosa on multiple sites, weekly for 6 weeks. Patients of group B were administered Injection PRP (2ml) weekly for 6 weeks along the same sites. The Maximum Inter-incisal Distance was previously measured with Xceptional Choice Digital Vernier Caliper initially, before starting the treatment and eventually on every visit. After completion, all treated patients were followed up monthly for 10 months. Examination of symptomatic subjective improvement was done and accurate documentation of Maximum Inter-incisal distance was carried out in all visits.

## Results:

A total of 44 patients were included in our study and divided randomly into two groups; 'A' and 'B', consisting of 22 patients each. Overall, there were 36 males and 8 females in the total study group. The male dominancy was evident in almost the same ratio in both groups; In Group 'A' the age ranged from 17 to 57 years and in Group B the age ranged from 22 to 55 years. All patients had a positive history of using areca nut ('Supari') either alone or in different combinations including Gutka and Paan. In group 'A' the mean pre-treatment Maximum interincisal distance (MIID) was 24.45mm, while in group 'B', it was 25.68 mm. After completion of 6 weeks of treatment, the mean MIID improved in group 'A' to 32.66 mm and in group 'B' to 34.73 mm. After the completion of the 6-month treatment, the mean MIID improved in group 'A' to 35.39 mm and in group 'B' to 36.13mm. The mean improvement in MIID after 6 months in group 'A' was 35.39+/-7.57mm compared to 36.13 +/-5.36 mm in group 'B'. The resultant P value in 1stweek of treatment is 0.461 and after 6 weeks is 0.276 and after 6 months is 0.710 which is statistically significant (Table 1) and Figure 1 (see PDF).

## Discussion:

Over the last 20 to 30 years some gutkha merchants took the help of actors from television shows to promote their areca nut product to the Indian population. With time slowly-slowly the paan and gutakha word is added very smartly with a common man's prestige. Television ads and the revolution of the internet give the chance to catch the maximum number of younger customers who use direct Areca Nut or any product manufactured by Areca Nut, eventually increasing oral submucous fibrosis cases [15]. There are a variety of non-surgical ways to manage OSMF. They include ayurvedic remedies, corticosteroids, proteolytic enzymes, vitamins, levamisole and placental extract [16]; numerous studies have utilized hyaluronidase with different combinations of short-, intermediate or long-acting steroids [17]. A survey of research that have been published generally shows that male predominate [18, 19-20] except for one research that produced conflicting findings [16] that study conducted in Karachi Pakistani population [21]. We findings are 4.5:1 in our study. According to research from Allahabad, India, where 46% of patients were in their third decade of life, areca nut product users are often young people [7] this is consistent with our study's findings, whereby the mean age was 35.14 years. Triamcinolone gives a better result than the injection of Placental extracts [23] and dexamethasone [24] but failed by other preparations like Lycopene [26] and colchicines [25]. The combination of lycopene, intralesional steroids and hyaluronidase is highly effective in enhancing mouth opening and alleviating other symptoms in patients with Oral Submucous Fibrosis [30]. A novel strategy for tissue regeneration called platelet-rich plasma (PRP) is emerging as a useful tool for accelerating healing in various kinds of medical and surgical operations. In 1987 M. Ferrari first time used PRP during open-heart surgery [27]. Whereas, PRP is most commonly observed to be used beneficially in soft and hard tissue healing [22]. Nowadays, it is often utilized in a variety of medical and surgical specialties, like general surgery, plastic surgery, neurology, urology, gynecology and dermatology. In 1997 First time performed the utilization of PRP in oral and maxillofacial surgery. This biological therapy copies the natural processes of wound healing by directing the whole protein show of plasma rich in growth factors to the injury site to repair the injured tissues. As a result, it promote encourages fibroblast proliferation and enhances tissue vascularity [28].

PRP is no longer content it only has a platelet concentration above the baseline. The normal counts of platelets in human blood are approximately between 150,000/µl and 350,000/µl and average about 200,000/µl but according to scientific proofs PRP has up to 1,000,000 platelets/µl locally, in a 5-ml volume of plasma [29]. Consequently, the concentration of platelets and growth factors is increased by 5 to 10 times over average. Up to 10 ml of the patient's blood is taken and sent to the lab to be processed in a centrifuge machine. A topical anesthetic is used before injections; however, while being a basic treatment, some patients may experience some pain or a burning sensation where the needle was inserted. Due to autologous, there is no risk of allergic responses or the spread of infectious infections. A thorough review of medical journal articles shows that there is still not a study published either describing the use of PRP in treating oral submucous fibrosis or evaluating its impact with that of intralesional steroids. Even if there is no similar research, examining the findings of various studies may provide an overview of the improvement in Maximum inter-incisor distance (MIID). An increase in MIID of 4.3±0.8mm following six weeks of biweekly injections of 1.5ml of dexamethasone and 1500 I.U. of hyaluronidase [30]. 6±2 mm with twice-weekly injections of 1.5 ml of dexamethasone and 1500 I.U. of hyaluronidase for four weeks [18], after intralesional injection of hyaluronidase of eight weeks the mouth opening is 9.38mm [19], 3.13 mm of mouth opening after receiving weekly injections of 4 mg of dexamethasone and 1500 I.U. of hyaluronidase for 12 weeks [20] and last one after receiving 4 mg (1 ml) injection of betamethasone twice a week for 8 weeks, MIID showed a 3.9 mm improvement [26]. The largest improvement was 9.38mm as a result. This is parallel to the findings of our study, in which group 'A' observed a mean improvement in MIID of 35.39+/- 7.57 mm (Table 4) compared to group 'B''s 36.13 +/- 5.36 mm (Table 2 and Table 3).

## Limitations of the study:

Since the sample size of the current study is very small, the results cannot be taken as strong evidence and therefore, studies with a larger sample size are required in the future. The study might be impacted by local elements include treatment methods, illness incidence and patient demographics. Also, the trial lacked double-blinding, meaning that both the patients and the investigators who were giving the treatments were aware of which one each patient was getting. Due to the possibility that the expectations as well as beliefs of the patients and investigators may have impacted the results, this might have brought bias into the study. Plus, the study's 6-week period could not have been sufficient for evaluating the therapies' long-term efficacy. An extended length of follow-up may yield further insights into the long-term efficacy of the therapies. PRP is highly effective but its price, difficulty and effort of collecting blood every time and the waiting period until the blood is processed and centrifuged are all limiting considerations.

## Funding information:

There was no outside funding for this study.

## Informed consent:

All study participants gave written informed consent and patients gave their consent in writing so that this work could be published.

## Institutional review board:

This study used a 1:1 allocation ratio, single-center, parallel randomized controlled trial design. Patients who were admitted to the outpatient unit of Radiology and Oral Medicine at Banaras Hindu University in Varanasi, Uttar Pradesh, India, were the source of the data. The Institutional Ethical Committee granted ethical clearance (EC3454). The trial is registered under the CTRI/2022/10/046263 number. Written informed permission was acquired from each participant and/or their legal guardian before to involvement in the study, which was carried out in compliance with the principles of the Declaration of Helsinki.

## Figures and Tables

**Table 1:** Comparison of mean mouth opening among two groups

**Interval**	**Triamcinolone + Hyaluronic Acid (A)**		**Platelet Rich Plasma (B)**		**p-value**
	**Mean**	**SD**	**Mean**	**SD**	
1^st^	24.45	5.26	25.68	5.76	0.461
2^nd^	26.11	5.64	27.29	6.05	0.51
3^rd^	27.85	6.2	29.26	6.28	0.459
4^th^	29.64	6.16	31.15	6.29	0.426
5^th^	31.06	6.28	32.81	5.81	0.343
6^th^	32.66	6.54	34.73	5.84	0.276
1 month	33.97	6.68	36.8	5.62	0.136
3.5 months	35	6.71	36.9	5.57	0.313
6 months	35.39	7.57	36.13	5.36	0.71

**Table 2:** Mouth opening in two groups

**Interval**				
	**Triamcinolone + Hyaluronic Acid(A)**	**Platelet Rich Plasma (B)**	**SD**	**SD**
1^st^	24.45	25.68	5.26	5.76
2^nd^	26.11	27.29	5.64	6.05
3^rd^	27.85	29.26	6.2	6.28
4^th^	29.64	31.15	6.16	6.29
5^th^	31.06	32.81	6.28	5.81
6^th^	32.66	34.73	6.54	5.84
1 month	33.97	36.8	6.68	5.62
3.5months	35	36.9	6.71	5.57
6 months	35.39	36.13	7.57	5.36

**Table 3:** Patients excel format (Platelet-Rich Plasma)

				**MOUTH OPENING**					**Platelet Rich Plasma**			**MOUTH OPENING**			
	**NAME**	**AGE/SEX**	**OSMF GREAD**	**1^ST^**	**2^ND^**	**3^RD^**	**4^TH^**	**5^TH^**	**6^TH^**	**FALLO-UP**	**1 month, MO**	**3.5 month, MO**	**6 month, MO**	**BURNING SENSATION (VAS)**	**Result**	**Platelet count**
1	Raja Ram	30/M	Greag II	30mm	32.6mm	33.4mm	35.5mm	38.2mm	40.3mm	42.6mm	43.5mm	43.2mm	85% Relif	3 to 5 mm in every week	1.5-4.10
2	Alok Singh	26/M	Greag III	21.1mm	19.5mm	21.2mm	23.5mm	24.3mm	26.8mm	28.6mm	28.3mm	28.6mm		No burning sensation after 1st injection	1.5-4.50
3	rambilashYadav	48/M	Gread III	25.8mm	28mm	30.2mm	33mm	35.5mm	36.8mm	40.2mm	41.7mm	40mm			1.5-4.30
4	PrashantSingh	22/M	Gread II	30.4mm	32.7mm	35.2mm	36.5mm	38.3mm	39.9mm	42.5mm	41.6mm	40mm			1.5-4.50
5	AkhileshYadav	25/M	Gread III	26mm	28.1mm	30.3mm	33.5mm	35.2mm	38.5mm	40mm	41.2mm	39.7mm			1.5-4.10
6	TabrejShekh	30M	Gread III	20mm	21.5mm	23.1mm	25.1mm	27.3mm	30.1mm	32.3mm	31.1mm	30.2mm			1.5-4.50
7	Subham Pal	22M	Greag III	20mm	21.7mm	22.5mm	23.8mm	25.1mm	27.1mm	30.1mm	30mm	29.5mm			1.5-4.50
8	Chandan Patel	27/M	Gread III	20mm	21mm	25mm	27mm	30.1mm	32.2mm	34.1mm	34mm	33.3mm			1.5-4.10
9	Anil Singh	28/M	Gread III	22.5mm	23.3mm	24.2mm	26.4mm	26.5mm	28.2mm	31.5mm	33.5mm	33.1mm			1.5-4.50
10	Vishal K. Singh	38/M	Gread II	30mm	31.5mm	32.2mm	34.1mm	35.8mm	37mm	40mm	41mm	40mm			1.5-4.30
11	Pawan K Singh	26/M	Gread III	23mm	24.4mm	26.5mm	28.5mm	29.5mm	31.5mm	33.2mm	33.5mm	33.1mm			1.5-4.50
12	PankajDube	32/M	Gread III	22.8mm	24.1mm	26.8mm	28.1mm	30.3mm	32.1mm	33.3mm	34.1mm	33.8mm			1.5-4.10
13	RekhaYadav	44/F	Gread III	24mm	26mm	27.6mm	29.3mm	31.2mm	32.5mm	34.1mm	33.5mm	32.7mm			1.5-4.50
14	Sudhir Kumar	38/M	Gread III	22mm	24.2mm	25.8mm	27.2mm	29.1mm	30.8mm	32.7mm	33mm	32.8mm			1.5-4.50
15	Mahesh K	28/M	Greag III	28mm	30.2mm	31.1mm	33.7mm	35.7mm	38.3mm	40.6mm	41.5mm	41.2mm			1.5-4.50
16	S.K. Soni	55/M	Greag III	20 mm	22.5mm	25.2mm	26.8mm	28.3mm	28.8mm	29.3mm	29.3mm	28.9mm			1.5-4.10
17	Hemlata	29/F	Gread II	33mm	40mm	43.2mm	44mm	44.5mm	45.8mm	46 mm	45.7mm	45mm			1.5-4.50
18	Meeradevi	38/F	Gread II	30.4mm	32.7mm	35.2mm	36.5mm	38.3mm	39.9mm	42.5mm	41.6mm	40mm			1.5-4.30
19	KaushalPandey	35/M	Gread III	28 mm	29.4mm	31.3 mm	33.6 mm	36.2mm	39 mm	42 mm	41.2mm	40 mm			1.5-4.50
20	SaurabhShukla	36M	Gread III	22 mm	23.5mm	25.2 mm	26.1mm	28.8mm	30.6 mm	32.8mm	33.1mm	31.2mm			1.5-4.10
21	Naveen Kumar	42M	Greag II	32mm	33.7mm	34.5mm	37.8mm	39.5mm	42.4mm	43.3mm	43.4mm	43.1mm			1.5-4.50
22	Neeru	35/F	Gread III	21mm	22.7mm	25mm	27.2 mm	30.1mm	31.4 mm	33.8mm	34mm	33.5 mm			1.5-4.50

**Table 4:** Patients excel format (Triamcinolone + Hyaluronic Acid)

				**MOUTH OPENING**					**Triamcinolone + Hyaluronic Acid**			**MOUTH OPENING**	
	**NAME**	**AGE/SEX**	**OSMF GREAD**	**1^ST^**	**2^ND^**	**3^RD^**	**4^TH^**	**5^TH^**	**6^TH^**	**FALLO-UP**	**After 1 month**	**After 3.5 month**	**After 6 month**	**BURNING SENSATION (VAS)**
1	Akhilesh K Singh	39/M	Gread III	20mm	21mm	21mm	23mm	24mm	24.5mm	24mm	25mm	26mm	no burning sensation after 1st injection
2	Anil K Yadav	32/M	Gread III	24.8 mm	25mm	27.5mm	30.2mm	32.4mm	34.8mm	36mm	37.6mm	37mm	
3	ARUN KUMAR	40/M	Gread III	22mm	24mm	25.3mm	26.7mm	28.3mm	30.2mm	30.2mm	32.6mm	31.7mm	
4	Khuddush Ahmad	17/M	Gread III	20mm	21.5mm	23.3mm	25.2mm	26mm	28mm	30.2mm	30mm	28.7mm	
5	Sudhanshu K. Singh	39/M	Gread III	20.5mm	21.8mm	22.5mm	23.4mm	23.8mm	25.1mm	26.5mm	28mm	29.2mm	
6	Seema	32/F	Gread III	20mm	20.5mm	21.6mm	24mm	24.7mm	26.2mm	27.4mm	27.5mm	26.7mm	
7	KamaleshUpadhyay	47/M	Gread III	21mm	23mm	24.7mm	26.2mm	27.8mm	28.5mm	29.2mm	28.9mm	28.5mm	
8	AmanBahadur	31/M	Gread III	20mm	21mm	21mm	23mm	24mm	24.5mm	24mm	25mm	26mm	
9	Amareshdube	33/M	Gread III	25mm	26.4mm	28.7mm	30.3 mm	32.4 mm	34.5mm	36.4 mm	37mm	37mm	
10	Alka Singh	29/F	Gread II	31.3 mm	32.3 mm	34 mm	36.3mm	37mm	39.1mm	41mm	41.6mm	41.5mm	
11	Anubhav	42/M	Gread III	25mm	27.1mm	30.3mm	31.7mm	33.3mm	35.2mm	37.2mm	37.6mm	37.5mm	
12	AlokRanjan	29/M	Gread III	20.5mm	21.mm	23.3mm	25.2mm	26.9mm	28.8 mm	30.2mm	32mm	32mm	
13	Ziledar	35/M	Gread III	20.1mm	21.5mm	21.9mm	23.4mm	23.8mm	25.1mm	26.5mm	28mm	29.2mm	
14	Shyamsundar	37/M	Gread III	20.7mm	21.5mm	22.8mm	24.2mm	25.7mm	27.2mm	28.9mm	30.5mm	30mm	
15	Shashikala	42/F	Gread III	23mm	25mm	27.3mm	29.5mm	31.6mm	32.5mm	34.7mm	34.5mm	33.5mm	
16	Suraj Patel	38/M	Gread III	22mm	23.4mm	25.1mm	27.7mm	30.7mm	33.1mm	35.6 mm	38.5mm	37.2mm	
17	Rakesh	28/M	Greag III	29 mm	31.2mm	32.1mm	34.7mm	36.7mm	38.6mm	40.5mm	41mm	41mm	
18	Hemant	45/M	Greag III	21 mm	23.5mm	25.3mm	26.8mm	28.4mm	30mm	32.8mm	33.5mm	32.9mm	
19	Kalyani Devi	57/F	Gread II	33mm	33.5mm	35.2mm	37mm	38.5mm	39.8mm	41mm	42.7mm	42mm	
20	Santosh	38/M	Gread II	30.4mm	32.5mm	34.2mm	36.5mm	38mm	39.9mm	41.5mm	41.6mm	41mm	
21	Ram prakash	42/m	Gread II	30.2mm	32.8mm	33.5mm	35.5mm	37.3mm	39mm	40.2mm	41.9mm	42mm	
22	Sunil Viswakarma	40/M	Gread II	33.3mm	34.5mm	36.2mm	38.5mm	40mm	41mm	41.5mm	42mm	42.5mm	

## References

[1] Rajendran R (1994). Bull World Health Organ..

[2] Ahmad M.S (2006). J Indian Soc Pedod Prev Dent..

[3] Trivedy C.R (2000). J Oral Pathol Med..

[4] Hazarey V.K (2007). J Oral Pathol Med..

[5] Pandya S (2009). Head Neck Oncol..

[6] Ceena D.E (2009). Indian J DentRes..

[7] Patil S, Maheshwari S (2014). J Clin Exp Dent..

[8] Arakeri G, Brennan P.A (2013). Br J Oral Maxillofac Surg..

[9] Kerr A.R (2011). Oral Dis..

[10] Albanese A.B (2013). Immun Ageing..

[11] Lin S.L (2018). Medicine (Baltimore)..

[12] Marx R.E (2004). J Oral Maxillofac Surg..

[13] Kevy S.V, Jacobson M.S (2004). J Extra Corpor Technol..

[14] Dhurat R, Sukesh M.S (2014). Journal of Cutaneous and Aesthetic Surgery..

[15] Dyavanagoudar S.N (2009). J Cancer SciTher..

[16] Yoithapprabhunath T.R (2013). J Pharm Bioallied Sci..

[17] Chole R.H, Patil R (2016). Intl J Contemp Med Resr..

[18] James Let (2015). J Int Oral Health..

[19] Gupta M (2014). Bangladesh Journal of Dental Research & Education..

[20] Yadav M (2014). J Oral Biol Craniofac Res..

[21] Shaikh A.H (2019). Professional Med J..

[22] Chandrakapure D (2023). Indian Journal of Otolaryngology and Head & Neck Surgery..

[23] Naik S.M (2012). Int J Head Neck Surg..

[24] Patel T.L, Singh S (2015). J Cont Med A Dent..

[25] Daga D, Singh R.K (2017). Natl J Maxillofac Surg..

[26] Singh D (2020). J Indian Acad Oral Med Radiol..

[27] Ferrari M (1987). Int J Artif Organs..

[28] Marx R.E (1998). Oral Surg Oral Med Oral Pathol Oral Radiol Endod..

[29] Marx R.E (2001). Implant Dentistry..

[30] Selvam N.P (2013). Asian J Pharm Clin Res..

